# Effect of the Nano Crystal Size on the X-ray Diffraction Patterns of Biogenic Hydroxyapatite from Human, Bovine, and Porcine Bones

**DOI:** 10.1038/s41598-019-42269-9

**Published:** 2019-04-11

**Authors:** Sandra M. Londoño-Restrepo, Rodrigo Jeronimo-Cruz, Beatriz M. Millán-Malo, Eric M. Rivera-Muñoz, Mario E. Rodriguez-García

**Affiliations:** 1 Posgrado en Ciencia e Ingeniería de Materiales, Centro de Física Aplicada y Tecnología Avanzada, Universidad Nacional Autónoma de México, Campus Juriquilla, 76230 Querétaro, Qro. Mexico; 2 Licenciatura en Tecnología, Centro de Física Aplicada y Tecnología Avanzada, Universidad Nacional Autónoma de México, Campus Juriquilla, 76230 Querétaro, Qro. Mexico; 3 Departamento de Nanotecnología, Centro de Física Aplicada y Tecnología Avanzada, Universidad Nacional Autónoma de México, Campus Juriquilla, 76230 Querétaro, Qro. Mexico

## Abstract

This paper focuses on the study of the effect of the change of the crystal size on the shape and width of the X-ray diffraction patterns for defatted and deproteinized bones as well as incinerated biogenic hydroxyapatite obtained from bovine, porcine, and human bones. Inductively Couple Plasma showed the presence of some ions such as Mg, K, Al, Fe, Zn, and Na for all samples. The nanometric size of the crystals was determined through High Resolution Transmission Electron Microscopy in which ordered crystals were found. The calcination of raw clean bones at 720 °C produced a transition of crystal size from nano to micro due to a coalescence phenomenon, this was accompanied by a decrease of the peak width of the X-ray diffraction patterns due to the decrease of the inelastic scattering contribution from the microcrystals. A simulation of the effect of the crystallite size on the shape and width of the X-ray patterns was done using PDF-4 software which confirmed that raw ordered bone crystals produce broad peaks which so far have been erroneously assigned to polycrystalline hydroxyapatite with low crystalline quality.

## Introduction

Nowadays, there is an increasing interest in nanomaterials in different fields such as tissue engineering. One of these is the hydroxyapatites (HAp) from biological sources such as human, bovine, and porcine due to their different applications^1–3^. The structure of these biogenic materials has been studied using X-ray diffraction (XRD). However, there is still a problem in the interpretation of their patterns concerning to the shape and width for raw and incinerated biogenic hydroxyapatite that are commonly used in clinical applications. This misinterpretation could limit their potential uses.

It is well established that the above-mentioned biogenic sources are formed by nanocrystals of HAp that contains minor elements such as Mg, Na, S, and K. Even with the presence of these ions, the XRD patterns of the raw bone mineral phase almost exhibit the same positions as those of synthetic hydroxyapatite^4^. But, the peaks for biogenic hydroxyapatites (BIO-HAps) are less sharp and broader which is attributable to the small size of crystals^5^. The ions presence influences the structural, thermal, optical, and morphological properties of hydroxyapatite^6^, and are very important for tissue engineering applications. Until now, several methods are widely used to isolate the mineral phase from the organic matrix of the bone: alkaline or acid hydrolysis, subcritical water process, and thermal decomposition (incineration)^7^.

The physicochemical properties of the isolated phase obtained by incineration depend on variables such as heating rate, annealing temperature, sintering time, cooling, and the atmosphere^6,8,9^. One of them is the crystal size that is commonly determined through X-ray diffraction. In the case of tissue engineering, some requirements are necessary for replacement materials among bioactivity, biocompatibility, biodegradability, osteoinductivity, enough mechanical properties, and a suitable architecture^10^. However, the nanometric crystal size as a requirement has not been explored. In this direction, it was found that some commercial hydroxyapatites for clinical applications showed microcrystals that could be the result of high annealing temperatures^4^.

The structural properties of hydroxyapatite from several sources have been studied by X-ray diffraction, in which information about the determination of crystalline structures, crystalline quality, crystal size, and lattice defects can be obtained. However, according to Scardi^11^ and Piga *et al*.^12^, the XRD analysis and the reliability of the results when the domain size is in the nanoscale as is the case of BIO-HAp, are still an open problem due to the elastic and inelastic signals are detected at the same time and affect the peak width and shape of the patterns.

Ooi *et al*.^13^ showed that the X-ray pattern of raw bovine bone exhibits the presence of nanocrystalline apatite. Their results indicate that in annealed samples between 700 to 1000 °C, a substantial increase in the height and a decrease in the width of the peaks occur which were associated with an increment in crystallinity and crystallite size. The same findings were reported by Niakan *et al*.^14^. However, if the initial state of the hydroxyapatite of the bovine bone corresponds to nanocrystals, the width and the height of the peaks cannot be correlated with crystalline quality due to elastic and inelastic scattering phenomena governing them.

Ramesh *et al*.^15^ studied the effect of the incineration process used to obtain HAps on their structure and found out that if the temperature increases, the crystalline quality follows the same trend, resulting in a change from broad to sharp peaks in the patterns. The average crystal size for nanoparticles is usually obtained by calculating the Full Width at the Half Maximum (FWHM) and the Scherrer’s equation as well as its modifications^16^. However, this equation has limitations since the lattice strain contributes to line broadening and the presence of organic components contribute strongly to the background. Both facts produce an error in the peak fitting and width calculation estimated in 10–15%^17^. Therefore, XRD patterns do not give a precise calculation of the crystal size. It only provides an order of magnitude for nanoscale-sized crystals in bones.

The crystallinity value is a parameter used to determine the atomic order of the atoms into the lattice and can be determined using an order parameter^18^ which can be affected by intrinsic and extrinsic factors. On the other hand, the crystalline quality (CQ) is a parameter that can only be determined through the FWHM when the pattern is governed by the elastic contribution. If the crystal size is in the range of some wavelengths as is the case of λ = 1.5406 Å for CuK_α_, it is not possible to determine the CQ because the pattern has elastic and inelastic contributions that are so far impossible to separate^19^.

Patel *et al*.^20^ using TEM images reported an average length of 48.58 ± 0.13 nm and a width 5.41 ± 0.09 nm for bovine bone, but they pointed out that this sample is amorphous. But their TEM images showed ordered crystals. Barakat *et al*.^21^ also studied the crystallinity of apatite from bovine bones using TEM images. However, the crystal size was not evaluated, and the crystalline percentage again was correlated with the peak width.

Considering the above-mentioned works, the objective of this paper was to study the effect of the change of the crystal size on the shape and width of the X-ray diffraction patterns of Bio-HAp from human, bovine, and porcine bones. The obtained results aim to clarify some misunderstandings regarding the interpretation of their diffraction patterns as well as some aspects related to the crystalline quality and crystal size and in this way to potentiate their uses in tissue engineering applications.

## Materials and Methods

### Raw bone and annealed samples

**Figure 1:**
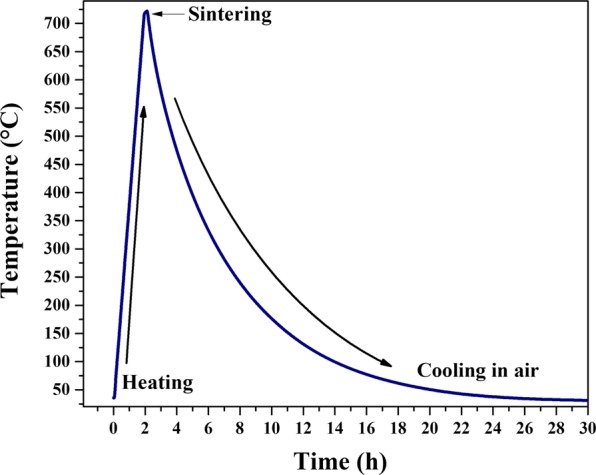
Thermal history for powder bones calcinated at 720 °C: B-720, P-720, and H-720.

Bovine, porcine, and human bones samples from femurs were used in this work. Human femur without apparent pathologies was donated by the Universidad Autónoma de Querétaro, femurs from bovine and porcine were collected from the local slaughter house. Cortical bone samples from each source were defatted and deproteinized following the methodology proposed by Londoño-Restrepo *et al*.^6^ to obtain clean bone powders. Each powder was sieved in a US 200 mesh (75 µm). These samples were dried and labeled as B-Raw for bovine, P-Raw for porcine, and H-Raw for the human. A fraction of each sample was simultaneously calcinated in a Felisa (Mexico) furnace at 720 °C and 6 °C/min heating rate for 10 min and cooled in furnace air according to thermal profile showed in Fig. 1. These last samples were labeled as B-720 for bovine, P-720 for porcine, and H-720 for human calcined at 720 °C. Synthetic hydroxyapatite from Sigma Aldrich (No. 28,939-6) was used for comparative purposes.

### Mineral content: ICP

The mineral composition of the raw and calcined samples as well as Sigma HAp was determined using the methodology proposed by Londoño-Restrepo *et al*.^6^ using a Thermo Fischer Scientific ICAP 6000 Series equipment with an argon plasma.

### TEM and SEM characterization

A high-resolution transmission electron microscope (S) TEM (JEOL ARM200F) was used to determine the crystal size of the BIO-Haps samples by using Image J free version, and images were processed using the Digital Micrograph software V4.0 from Gatan and the array mask was applied to clean the FFT signal. SEM images of the incinerated samples were obtained using a High-Resolution Scanning Electronic Microscope (SEM) model SU8230(Hitachi).

### X- ray diffractions of bone

X-ray diffraction technique was used to obtain the patterns of synthetic HAp from Sigma Aldrich, clean bone powders B-Raw, P-Raw, and H-Raw, and calcined samples B-720, P-720, and H-720. The FWHM for all these samples was used to evaluate the effect of the crystal size on the XRD patterns. Patterns were obtained using a Rigaku Ultima IV diffractometer operating at 35 kV, 15 mA with CuK_α_ radiation wavelength of λ = 1.5406 Å. Diffractograms were taken from 5 to 80° in a 2θ scale and 0.02° step size for phase identification. The effect of the crystal size on the shape of the diffraction patterns was analyzed by a simulation done using PDF-4 software by varying the crystallite size remaining a perfect order in the crystalline structure.

## Results and Discussion

### Mineral composition

**Figure 2:**
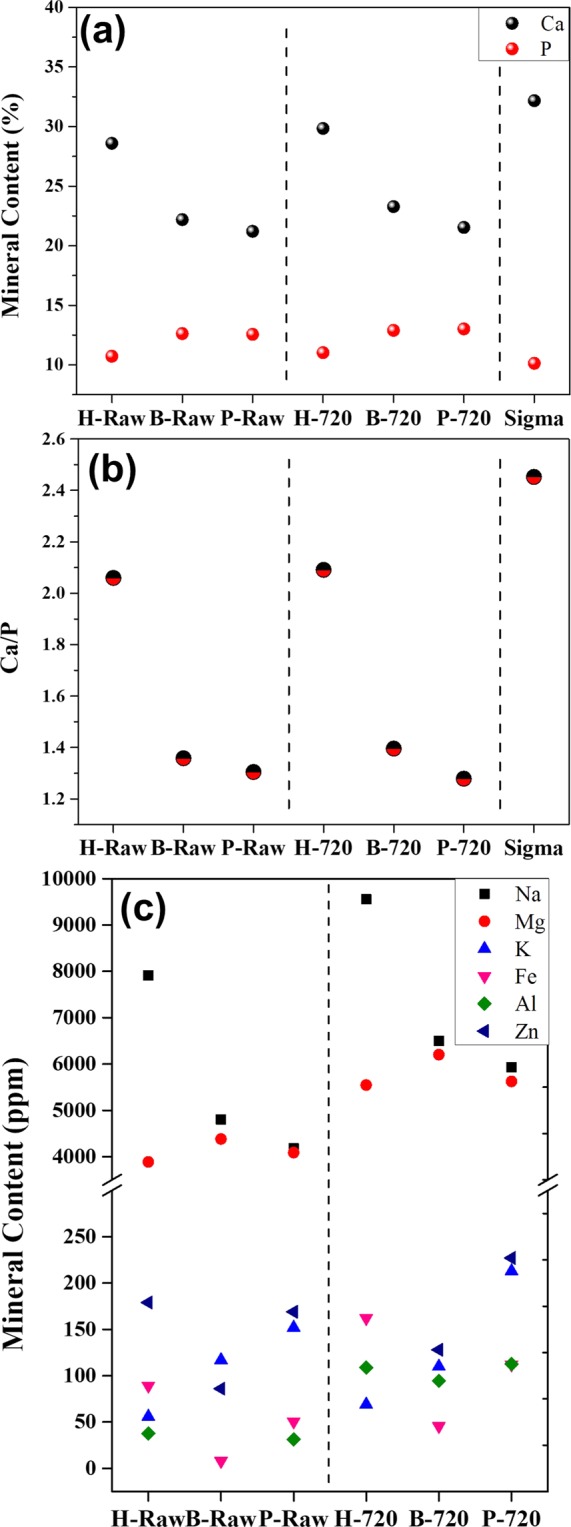
(**a**) Ca and P content for HAp from Sigma, raw, and sintered samples, (**b**) Ca/P ratio, and (**c**) minority mineral composition.

Figure 2(a) shows the mineral content of Ca and P majority elements for raw and sintered BIO-HAp samples, and HAp from Sigma. No differences between raw clean bones and samples calcinated at 720 °C were found. Ca levels are higher for human than for bovine and porcine bone; it could be due to the human diet rich in calcium, and that this HAp is carbonated. The high Ca content in H-Raw increases the Ca/P ratio (see Fig. 2(b). After calcination, this ratio does not suffer any change because samples were defatted and deproteinized before the process. Sigma-Aldrich sample showed the highest Ca content; thus, its Ca/P ratio is higher than for pure hydroxyapatite. Figure 2(c) exhibits the content of the mineral traces in the bones which is well known to enhance bone regeneration. The presence of these elements depends on several factors such as diet, age, gender, among others. It is also remarkable that the incineration does not affect the mineral content, and the small variations in these values correspond to a concentration effect^6^. Minoritarian elements can be located as substitutional or interstitial atoms that can change their structure.

### TEM analysis

**Figure 3:**
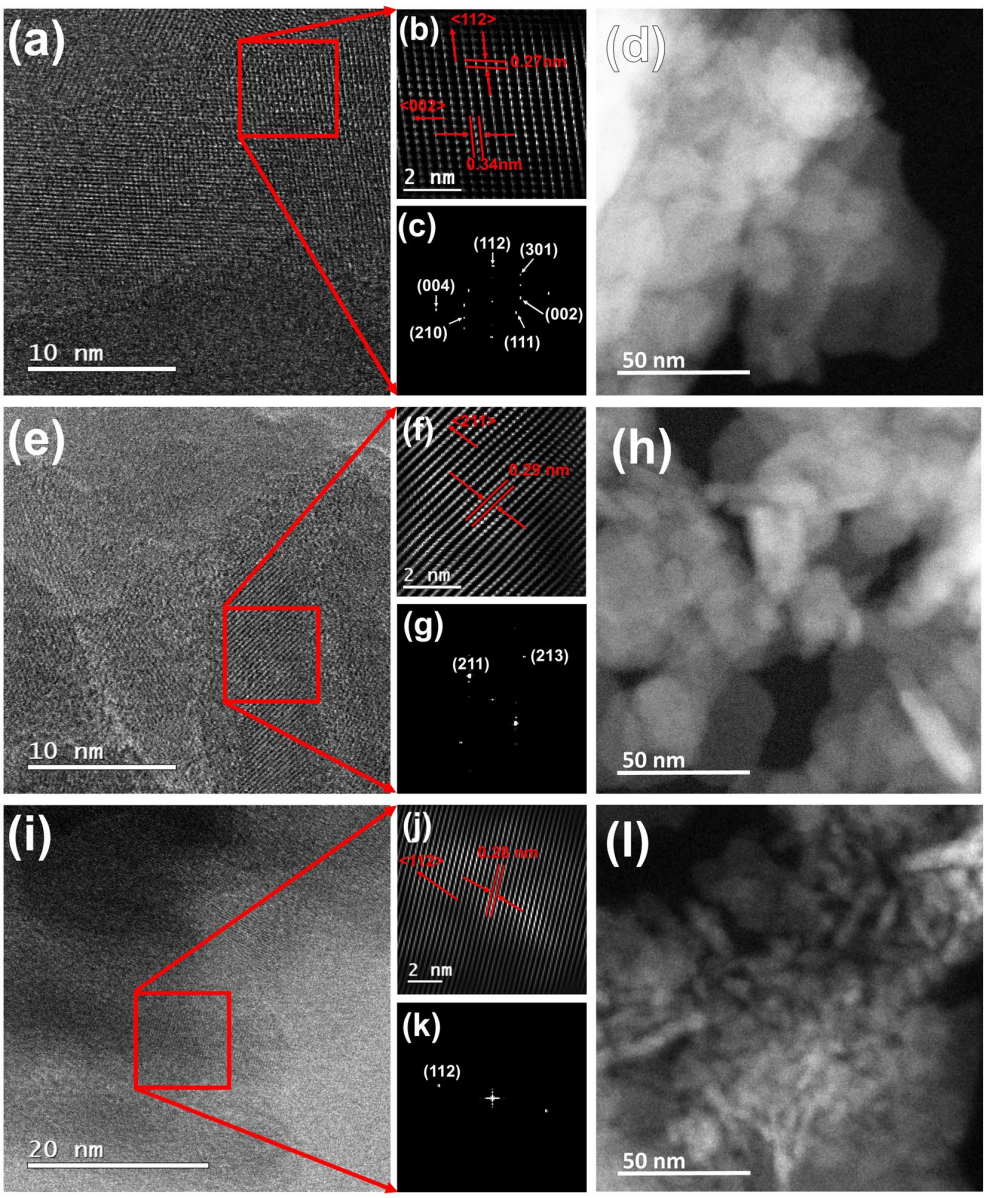
HRTEM images for raw HAp crystals: B-Raw (**a–d**), P-Raw (**e–h**), and H-Raw (**i–l**).

Figure 3 shows HRTEM images for defatted and deproteinized B-Raw (a–d), P-Raw (e–h), and H-Raw (i–l) samples, where B-Raw corresponds to defatted and deproteinized bovine bone powder, P-Raw for porcine, and H-Raw for human. HRTEM images showed that clean raw bones are formed by polycrystalline nanocrystals with elongated plate shapes. Xin *et al*.^22^ reported HRTEM images of cortical human bones as thick mineral flakes and needles. However, in our work, the needles structures for human bone were not found, but their interpretation can be based on the transversal view of the elongated plates or flakes.

The Image J software was used to determine the crystal size (2D) and the average value for 25 determinations were: 21 ± 8 nm and 6 ± 1.5 nm for H-Raw; 13 ± 3 nm and 7 ± 2 nm for B-Raw; and 17 ± 4 nm and 6.4 ± 0.4 nm for P-Raw, for length and width, respectively. These findings confirm the nano-dimensions of the hydroxyapatite crystals of raw bones, which are elongated like-plates that can be correlated with the well-known longitudinal and preferential growing of the bone^6^. TEM analysis showed the presence of well-defined nanocrystals and the (112) and (002) planes for bovine bone, (211) for porcine, and (112) for human, indicating crystalline order. Patel *et al*.^20^ pointed out that raw bone powder from bovine is crystalline based on TEM images. According to Glimcher^23^ longitudinal like plates are formed by small spherical ones that are the youngest nanocrystals and the long needle shaped ones that are the older crystals.

The determination of the distances between the lines and dots was done using the Digital Micrograph software. All these samples are polycrystalline because of the multiple directions that are observed in their Fast Fourier Transform images (Fig. 3(c), (g) and (k)). For B-Raw, some dots are separated by 0.27 nm which corresponds to the <112> , while the other distance (0.34 nm) corresponds to <002 > family of directions. In the case of P-Raw, the direction <211 > was identified as well as the <112 > for H-Raw nanocrystals. All these planes were identified using the ICDD card # 01-084-1998 for synthetic hydroxyapatite.

### Crystalline order through the TEM images

The atomic order showed for H-Raw, B-raw, and P-Raw in Fig. 3(a), (e), and (i) (see squares), clearly evidenced that the atoms that form these BIO-HAp are ordered into the hexagonal structure. These HRTEM images and the ones reported by other authors such as Xin *et al*.^22^ confirmed this fact. This finding indicates that it is necessary for a reinterpretation of the X-ray diffraction patterns for nanocrystals, and of course, there is a misunderstanding related to the poorly crystalline quality of these BIO-HAp^4,24–30^. Then, based on our analysis, it is not possible to define raw hydroxyapatites as structures with low crystalline quality.

### SEM: morphological analysis

**Figure 4:**
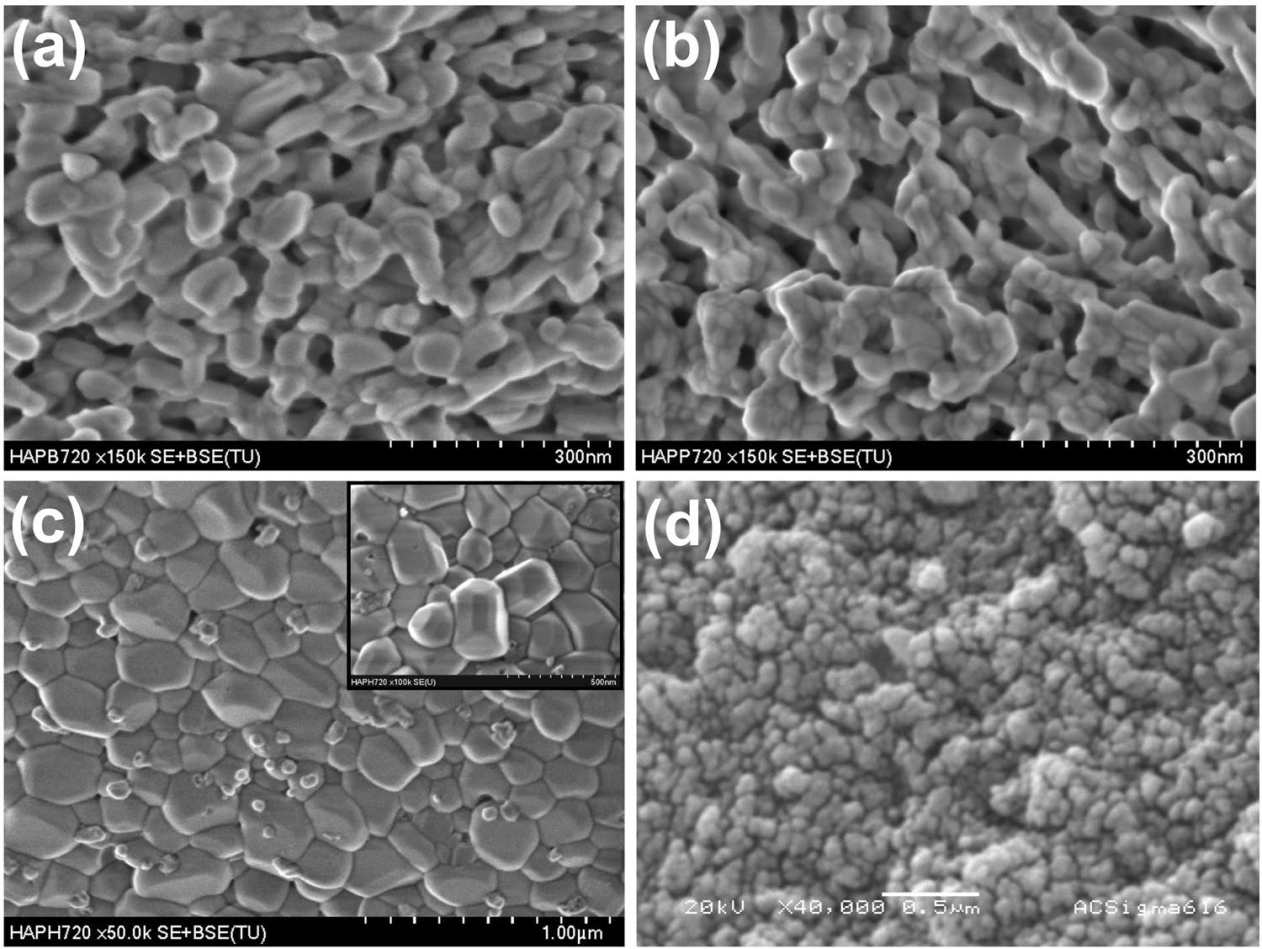
SEM images for calcinated samples: (**a**) B-720, (**b**) P-720, (**c**) H-720, and (**d**) Sigma-Aldrich.

After calcination at 720 °C, HAp crystals reach sizes in the order of microns as in the case of H-720. Figure 4 shows SEM images for calcinates samples B-720, P-720, and H-720. The B-720 sample exhibits crystals with elongated shapes whose boundaries are joined to those of other crystals. It evidences that the growing process by coalescence was interrupted (Fig. 4(a)). In the case of P-720 sample, a bunch of crystals can be seen, which allows determining that the elongated structures are formed by their coalescence (Fig. 4(b)). Human HAp crystals have irregular faceted shapes, and some hexagonal prisms are observed, but also small spherical crystals were identified as magnesium oxide as will be shown in the XRD section. It is worth noting that B-720 and P-720 are porous systems in which blood irrigation and vascularization are possible when these materials are used for clinical applications, but hydroxyapatite crystals for H-720 sample do not have any pores.

Again, the Image J software was used to determine the 2D dimensions of the HAp crystals. The average lengths and widths are 117 ± 19 nm and 42 ± 9 nm for B-720 and 62 ± 9 nm and 28 ± 4 nm for P-720, respectively. In these cases, BIO-HAps are not single crystals yet but coalesced crystals that are still growing; this is the reason for the huge standard deviation for both samples. On the other hand, H-720 crystals developed the highest sizes until 330 ± 60 nm length and 200 ± 40 nm width on average. Pseudo-spherical crystal shapes and some hexagonal structures with well-defined facets were found (see inset in Fig. 4(c)). The difference in the crystal sizes reached after the calcination process at 720 °C may be due to the crystal size of the raw samples. All raw crystals are nanometric, but smaller crystals as the human sample have a higher surface area that reacts, and then bonds are rearranged between the surrounding crystals. Hydroxyapatite from Sigma Aldrich exhibits spherical particles with 48 ± 4 nm with apparent interconnected porosity which means that its process of synthesis developed particles bigger than those for raw biogenic apatites and there no was a calcination process at elevated temperature involved in the synthesis (see Fig. 4(d)).

### X-ray diffraction

**Figure 5:**
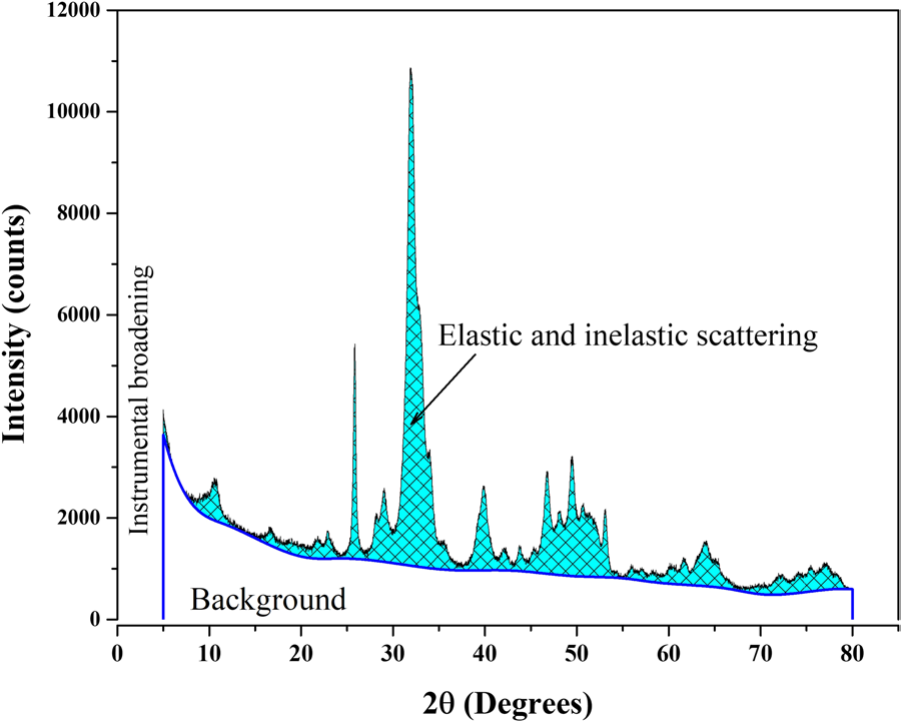
Typical X-ray diffraction pattern of defatted and deproteinized bovine bone.

Figure 5 shows a characteristic XRD pattern of a defatted and deproteinized cortical bovine bone. As it well known, a pattern is formed by: sample contribution (crystalline and amorphous), noise (background), and instrumental function. However, it is critical to show that the region that has been used to describe the crystalline contribution of a nanocrystalline sample is formed by elastic and inelastic scattering and the instrumental function, which means that it is not possible to determine independently each one of these signals. In this direction, there is a paper “Separating diffraction from scattering: the million-dollar challenge” by Laven^19^, that shows that this is an instrumental and physics problem in different areas where both contributions are present. Finally, the regions that can be defined in X-ray pattern of a nanoparticle sample are the elastic and inelastic scattering contributions that involve information regarding the nano-size character of the crystals in the sample, the amorphous region, and the background (see Fig. 5). Each instrument has an instrumental function that contributes to the final shape of the pattern. It means that the calculation of the crystalline percent cannot be obtained in the case of nanoparticles because so far it is impossible to separate the inelastic scattering contribution and the diffracted signal.

**Figure 6:**
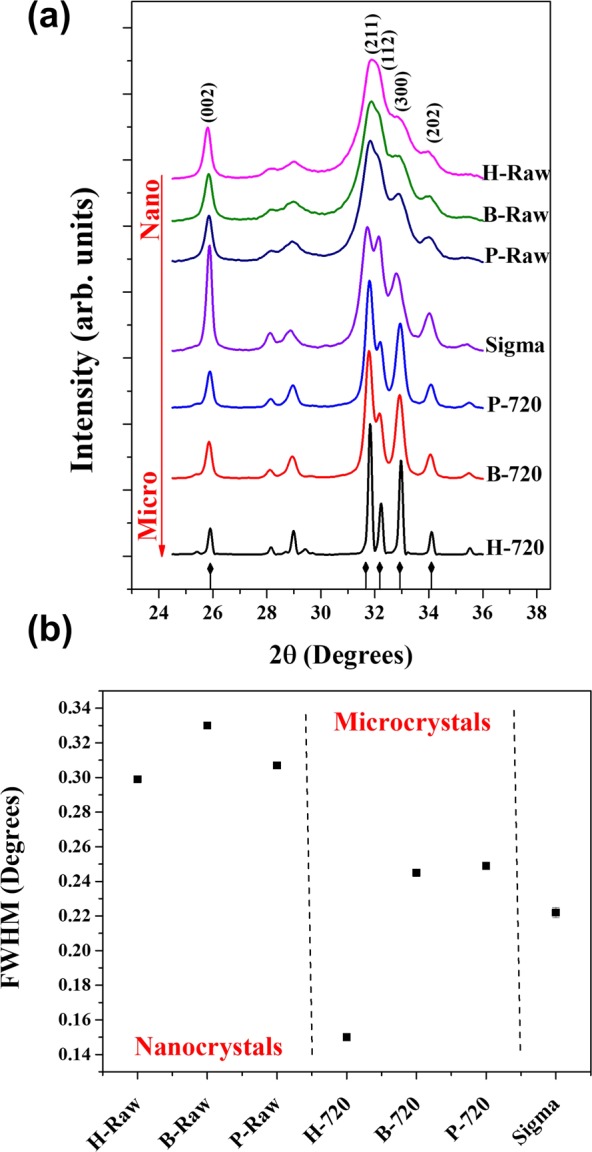
(**a**) X-ray diffraction patterns for all samples (H-Raw, B-Raw, P-Raw, H-720, B-720, P-720, and Sigma), and their respective (**b**) FWHM calculated for the (002) peak.

Figure 6(a) shows the X-ray patterns of nano-Bio-HAp obtained for H-Raw, B-Raw, and P-Raw bones, incinerate samples H-720, B-720, and P-720, and Sigma Aldrich. The patterns of raw Haps exhibit broad and not well-defined peaks and after calcination the H-720, B-720, and P-720 patterns are characterized by sharp and well-defined peaks.

The peaks identification in these X-ray patterns was done by ICDD card No. 01-084-1998 for hydroxyapatite. The peak width of the incinerated samples in relation to the raw samples decrease, while an opposite trend was found for the intensity, indicating that the elastic contribution governs the patterns for incinerated samples while the X-ray diffraction of raw materials has elastic and inelastic scattering contributions.

In terms of the XRD analysis, the patterns for raw HAps have been interpreted as polycrystalline samples with “low crystalline quality,” but according to the HRTEM images these samples correspond to ordered HAp nanocrystals. The changes in the crystalline quality (atomic order) are influenced by the inclusion of different ions into the lattice as substitutional or interstitial sites.

All raw samples and B-720, and P-720 were identified as hydroxyapatite (ICDD No. 01-084-1998), but for the H-720, other crystalline phases were identified by using the following power diffraction files: Whitlockite ICDD card # 00-009-0169, NaCaPO_4_ ICDD card # 00-029-1193, Calcite ICDD card # 00-047-1743, MgO ICDD card # 00-004-0829, CaO ICDD card # 00-037-1497. Additional phases present in the H-720 sample are reported in Table 1; MgO is confirmed in the SEM image for H-720 (Fig. 5(c)) as small spheres which were previously identified by Energy-Dispersive X-ray Spectroscopy (EDS)^31^. The presence of the additional phases for H-720 sample obeys to the age of the bone. It is well known that bovines are slaughtered 2-years-old and 5-months-old for pigs, while the studied human bone belonged to an adult that changes its composition due to metabolic activity, aging, and disease. Although, the presence of MgO is due to a diffusion issue where magnesium naturally present in the bone^32^ reaches the melting point at 650 °C and the oxidative atmosphere where the sample was calcined.

**Table 1:** Additional crystalline phases found for H-720.

Sample peak position 2θ (°)	Phase	Peak position 2θ (°)	(hkl)
26.08	NaCaPO_4_	26.21	(200)
28.69	NaCaPO_4_	28.68	(121)
29.42	CaCO_3_	29.40	(104)
29.71	Ca_3_(PO_4_)_2_	29.65	(300)
30.89	Ca_3_(PO_4_)_2_	31.02	(0 2 10)
31.98	NaCaPO_4_	32.09	(130)
32.38	Ca_3_(PO_4_)_2_	32.44	(128)
32.97	Ca_3_(PO_4_)_2_	33.02	(306)
33.12	NaCaPO_4_	33.14	(002)
33.45	Ca_3_(PO_4_)_2_	33.48	(1 1 12)
37.39	CaO	37.34	(200)
40.01	Ca_3_(PO_4_)_2_	40.06	(1 1 15)
40.23	Ca_3_(PO_4_)_2_	40.21	(042)
42.93	MgO	42.91	(200)
43.25	CaCO_3_	43.14	(202)
53.91	CaO	53.85	(220)
60.26	Ca_3_(PO_4_)_2_	60.37	(158)
62.44	MgO	62.30	(220)

One of the problems related to the interpretation of the XRD patterns of HAps is the use of the FWHM as a parameter to quantify their crystalline quality. But, by direct inspection of this parameter in raw samples, these peaks are broad. Based on the FWHM of a characteristic peak, low crystalline quality has been reported for BIO-HAp, and the calculation of the crystallinity percentage is carried out considering the complete XRD pattern. However, the broad peaks that the diffractograms display are due to the inelastic scattering originated from the BIO-HAp nanocrystals, the broadening originated by itself diffraction, and the instrumental contribution. It means that the calculation of the percent crystallinity and crystalline quality in nanocrystals must be revised in detail in the future.

As shown in Fig. 6(a), these patterns correspond to nanocrystalline samples. As it has been pointed out in different scientific works, hydroxyapatites from mammalian bones have been characterized as nanoparticles with low crystalline quality, considering that the diffracted peaks are broad and weak^4,24–30^. However, by the inspection of different HRTEM images for these samples, they have a crystalline order.

Figure 6(b) shows the FWHM for the (002) peak of XRD patterns for raw samples (H-Raw, B-Raw, and P-Raw), calcined samples (H-720, B-720, and P-720), and Sigma Aldrich. Raw samples showed higher FWHM values than the calcinated samples, which is not an indicative that these samples are poorly crystalline.

As was discussed above, HRTEM images show that the raw samples are formed by nanocrystals which mean that the XRD patterns for raw HAp correspond then to nanocrystals as well. Again, the broad peaks are mainly the result of a simultaneous elastic and inelastic scattering. On the other hand, for the H-720 the value of the FHWM is the lowest one. Here, the interpretation done by different authors is referred as that the FWHM decreases due to an increase of the crystalline quality of the sample, but the incineration process produces a coalescence phenomenon that originates the growth of the crystal and the X-ray diffraction instead inelastic scattering governs this kind of patterns^6^. It can be interpreted as follows: first, the raw crystals improved their crystalline quality or due to the heating process the raw samples suffer a coalescence prosses increasing the crystal size from nano to micro, but it cannot be interpreted saying that the crystalline quality of the sample increases as is commonly reported^9,13,33,34^.

### Simulation of the effect of the crystalline size on the XRD patterns

The effect of the crystal size on the X-ray diffraction patterns has been studied in detail by using the PDF-4 software^35,36^, that consider the peak shape analysis to provide information on particle size and strain distributions of the samples as well as the Bragg intensities that gives information about possible preferred orientation and texture effects in powder samples, for the PDF-4 analysis of the effect of the crystal size a characteristic Powder diffraction files for synthetic HAp (ICDD No. 01-084-1998) was used^37^.

**Figure 7:**
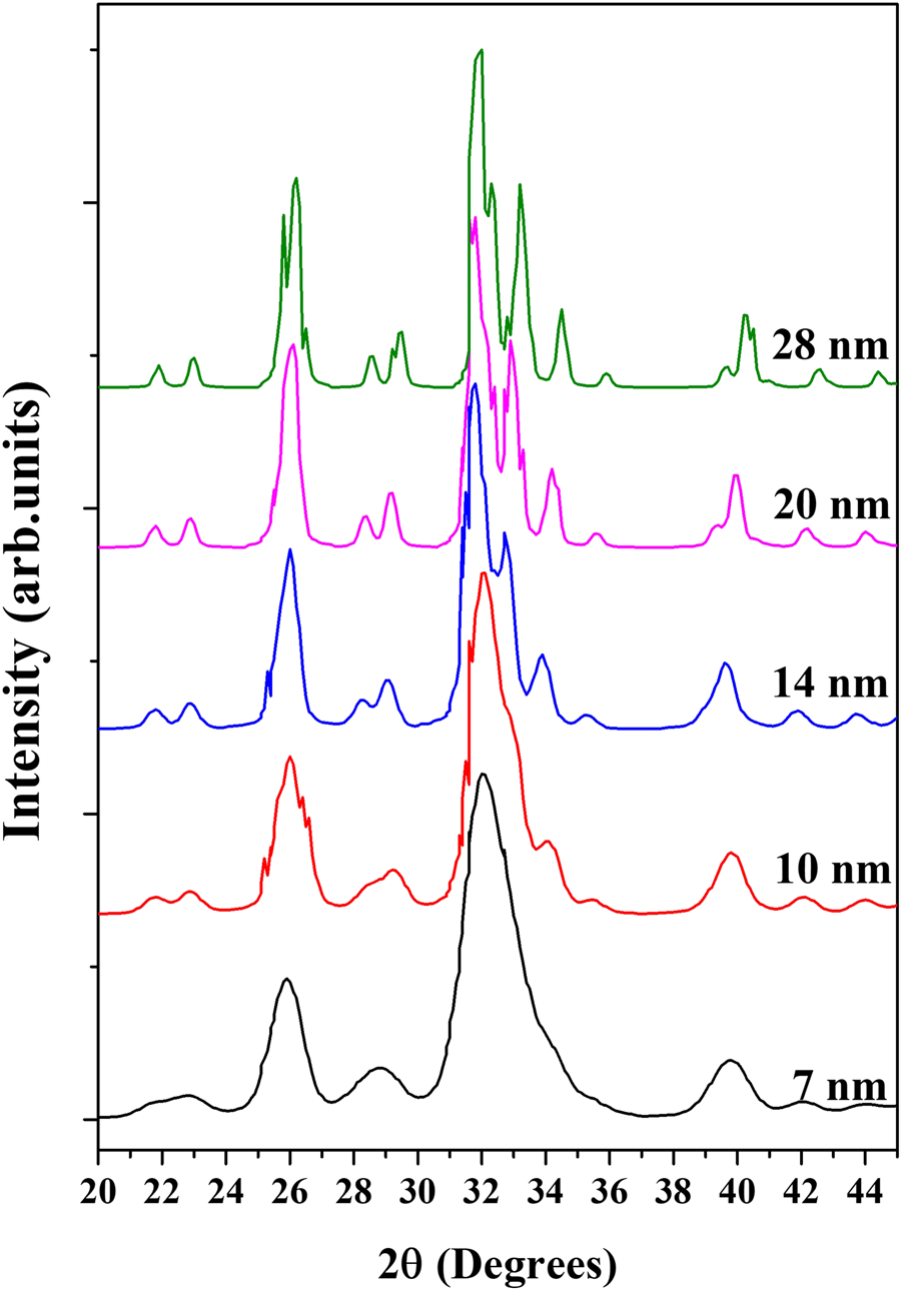
Simulated X-ray diffraction patterns of hydroxyapatite as a function of the crystal size.

As was confirmed by HRTEM images for the raw HAp samples, all of them are nanocrystals with lengths below 20 nm and width about 7 nm, and according to Fig. 6(a) nanocrystals produces drastic changes in the shape and width of the XRD patterns. As it was mentioned before, it is possible to simulate the effect of the crystal size on the shape and width of the XRD patterns using PDF-4 software and this software was considered an ordered crystal. For this simulation and considering the HRTEM findings, the initial crystal size was 7 nm and the effect of the growth of the crystals was evaluated. Figure 7 shows the simulation of the XRD patterns for HAp using the ICDD No. 01-084-1998 for HApvarying the crystal size from 7 to 28 nm. For crystal sizes of 7 and 10 nm, the patterns correspond to broad peaks. The crystal size is sometimes the incident wavelength of the CuK_α_ radiation, in this simulation, it was not considered the amorphous contribution of the material. While, if in the simulation, the crystal size increases from 14 to 28 nm, the broad peaks become narrower and more defined because the X-ray inelastic scattering decreases and the resulting diffracted peaks are governed by the elastic scattering (diffraction) in the crystalline structure of the hydroxyapatite crystals. Here, it is important to clarify that this effect is less evident for the studied samples due to other issues (instrumental, sample nature, among others) that influence the pattern. These simulations showed clearly that the reported XRD patterns for raw bones, in fact correspond to nanocrystals and these do not represent as it has been mentioned before “poly-crystals with low crystalline quality”.

With techniques as HRTEM or SEM it is possible to determine directly the crystal size. Another technique used in determining or estimating the crystal size of polycrystalline samples is X-ray diffraction using the Scherrer’s equation. However, it has some limitations, for example, derivation of Equation 1 does not consider the type or scattering power of the atoms, and instrumental function. These acceptable results are restricted to sizes up to a few hundreds of nanometers. Cullity and Stock^38^ showed that the Scherrer’s equation is valid only for crystallite sizes up to 200 nm and has close relation to the resolution of the diffractometers. According to Miranda and Sasaki^39^ in an X-ray diffraction pattern, there is a multiple scattering, the waves scattered by one atom (or atomic plane) are also scattered by the other atoms (or atomic planes) and those waves are also subsequently scattered by other atoms and so on. The limits of the Scherrer’s equation have been described by Muniz *et al*.^40^ suggesting that there is a limit of applicability of this equation and found a dependence on the Bragg angle and the absorption coefficient of the XRD pattern. However, their findings are not general.

1 $$CS=\frac{k\lambda }{\beta Cos\theta }$$

Where λ is the wavelength, k is a constant (0.9), β is the FWHM in radians, and θ is the diffraction angle. For this sample, β and θ were determined in the (002) peak. Scherrer equation is limited to crystal sizes from 100 to 200 nm. Calculate sizes smaller than this range by this equation has the problem of the separate peak broadening due to the crystallite size from the broadening due to other factors^41^. Crystal size of H-Raw, B-Raw, and P-Raw was calculated using Scherrer’s equation.

**Figure 8:**
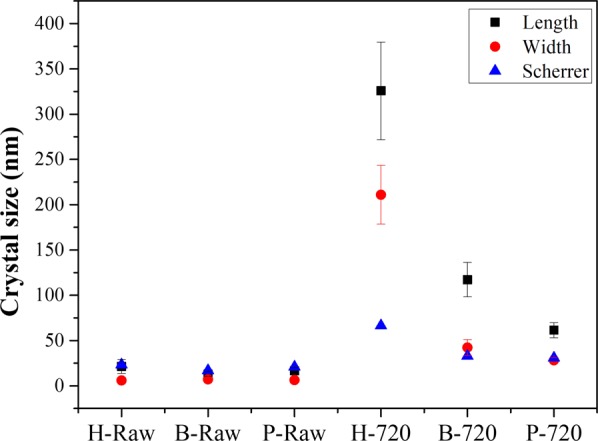
Crystallite sizes obtained by using Scherrer’s equation and by HRTEM and SEM images processing.

Figure 8 shows the crystal size of biogenic hydroxyapatites (raw and calcined) that was calculated by using the Scherrer’s equation using the FWHM value calculated for (002) peak, and by TEM and SEM images processing (ImageJ free software) for raw and calcined samples, respectively. The crystal sizes for raw samples obtained for both methods are consistent between them, which confirms that Scherrer’s equation is an excellent tool to determine the crystal size for nanocrystals, but in the case of micron crystals, the limitations of the equation do not allow determining their crystal size. Based on these values, it is clear that the HAp crystals suffered a dramatic change in their sizes which affected the shape of the X-ray diffraction patterns.

## Conclusions

The shape and width of the X-ray diffraction pattern for ordered crystals are governed by the crystal size. The broad peaks of the patterns for nano HAP are not necessarily related to disordered crystalline structures.

HRTEM images of the raw clean bones showed ordered structures, which means that the broad peaks of the X-ray patterns are originated by the simultaneous inelastic and elastic scattering produced by the beam/structure interaction. So far, the inelastic contribution cannot be separated from the signal produced by the elastic scattering that forms a diffraction pattern. HRTEM images showed in diluted samples that the needles are produced by transversal view of the elongated plates.

PDF-4 simulations of the effect of the crystallite size on the shape and width of the X-ray diffraction patterns confirm that both elastic and inelastic contributions in ordered BIO-HAp originated broad peaks.

This misinterpretation about the correlation between broad peaks and disordered crystals has caused the erroneous belief that hydroxyapatite nano crystals from mammalian are poorly crystalline, but as it was desaturate in this work, is that these nanocrystals are ordered as was shown with the HRTEM images.

The XRD pattern of nano crystals is governed simultaneously by elastic and inelastic scattering. The incineration process produces an increase in the crystal size, and a decrease in the FWHM value, which cannot be directly related with an improvement of the crystalline quality. It is the result of the transformation from nano to micro HAp crystals.

From a tissue engineering point of view, these results showed that incineration process can be applied to obtain BIO-HAp, but the XRD patterns must prove that the temperature does not originated changes in its nanometric size.
